# Establishing nonlinearity thresholds with ultraintense X-ray pulses

**DOI:** 10.1038/srep33292

**Published:** 2016-09-13

**Authors:** Jakub Szlachetko, Joanna Hoszowska, Jean-Claude Dousse, Maarten Nachtegaal, Wojciech Błachucki, Yves Kayser, Jacinto Sà, Marc Messerschmidt, Sebastien Boutet, Garth J. Williams, Christian David, Grigory Smolentsev, Jeroen A. van Bokhoven, Bruce D. Patterson, Thomas J. Penfold, Gregor Knopp, Marek Pajek, Rafael Abela, Christopher J. Milne

**Affiliations:** 1 Paul Scherrer Institut, Villigen, Switzerland; 2 Institute of Physics, Jan Kochanowski University, Kielce, Poland; 3 Department of Physics, University of Fribourg, Fribourg, Switzerland; 4 Department of Chemistry, Uppsala University, Uppsala, Sweden; 5 Institute of Physical Chemistry, Polish Academy of Sciences, Warsaw, Poland; 6 Linac Coherent Light Source, SLAC National Accelerator Laboratory, Menlo Park, USA; 7 NSF BioXFEL STC, 700 Ellicott Street, 14203 Buffalo, USA; 8 Brookhaven National Laboratory, Upton NY 11973 USA; 9 Institute for Chemical and Bioengineering, ETH Zürich, Zürich, Switzerland

## Abstract

X-ray techniques have evolved over decades to become highly refined tools for a broad range of investigations. Importantly, these approaches rely on X-ray measurements that depend linearly on the number of incident X-ray photons. The advent of X-ray free electron lasers (XFELs) is opening the ability to reach extremely high photon numbers within ultrashort X-ray pulse durations and is leading to a paradigm shift in our ability to explore nonlinear X-ray signals. However, the enormous increase in X-ray peak power is a double-edged sword with new and exciting methods being developed but at the same time well-established techniques proving unreliable. Consequently, accurate knowledge about the threshold for nonlinear X-ray signals is essential. Herein we report an X-ray spectroscopic study that reveals important details on the thresholds for nonlinear X-ray interactions. By varying both the incident X-ray intensity and photon energy, we establish the regimes at which the simplest nonlinear process, two-photon X-ray absorption (TPA), can be observed. From these measurements we can extract the probability of this process as a function of photon energy and confirm both the nature and sub-femtosecond lifetime of the virtual intermediate electronic state.

Since the introduction of optical lasers, high pulse energies and ultra-short pulses are commonly used to probe and modify in a controlled way the electronic states of matter^1^. At sufficient laser intensities, the nonlinear regime is reached where the interaction of the electro-magnetic field of the incident pulse with the electrons in the material leads to a variety of effects, which depend nonlinearly on the incident pulse energy. Presently, these nonlinear material responses find applications in a large variety of research fields, including fundamental atomic physics, quantum theory, as well as applied sciences^2^,^3^,^4^. However, due to the laser sources employed, these techniques have been generally limited to optical wavelengths (λ > 200 nm). Therefore, the nonlinear interaction of Ångstrom X-ray wavelengths with matter has remained an unexplored regime. The introduction of XFEL facilities that can produce high per-pulse photon flux (~10^12^) with short pulse durations (ranging from 2–50 femtoseconds) has provided the ability to reach very large peak powers in the keV regime^5^,^6^,^7^. This has permitted the first observations of processes with very low probabilities, including double-core hole formation^8^,^9^, two-photon absorption^10^,^11^, amplified spontaneous X-ray emission^12^,^13^, plasma creation^14^, X-ray-optical wave mixing^15^, X-ray second harmonic generation^16^ and anomalous X-ray Compton scattering^17^.

Among the different possible pathways for nonlinear X-ray interaction with matter, the two-photon absorption (TPA) mechanism possesses attractive features that may be explored by performing X-ray spectroscopy at XFELs. The TPA process was theoretically predicted in 1931^18^ and experimentally demonstrated in the 1960’s^19^,^20^ thanks to the development of optical laser sources, and is now commonly used as a tool in a plethora of scientific areas^21^,^22^,^23^,^24^. However, in the hard X-ray regime, the TPA process has only been observed in condensed matter for the first time very recently^10^ thanks to the development of XFEL sources. When applied at hard X-ray photon energies, the TPA mechanism leads to creation of new and unexplored electronic states of matter. In contrast to one-photon absorption (OPA) processes, which are determined by dipole-allowed transitions, the TPA process requires excitation between states of the same parity. The TPA selection rules lead to excitation of dipole-forbidden transitions, such as quadrupole or higher-order excitations. Moreover, contrary to optical wavelengths, the TPA mechanism at hard X-ray energies allow for element- as well as core-level selective excitations. Thus the TPA process, besides its possible application to quadrupole-allowed X-ray absorption spectroscopy, also allows the verification of the quantum description of the interaction of X-ray radiation with bound electrons in the nonlinear regime^25^.

## Results and Discussion

**Figure 1:**
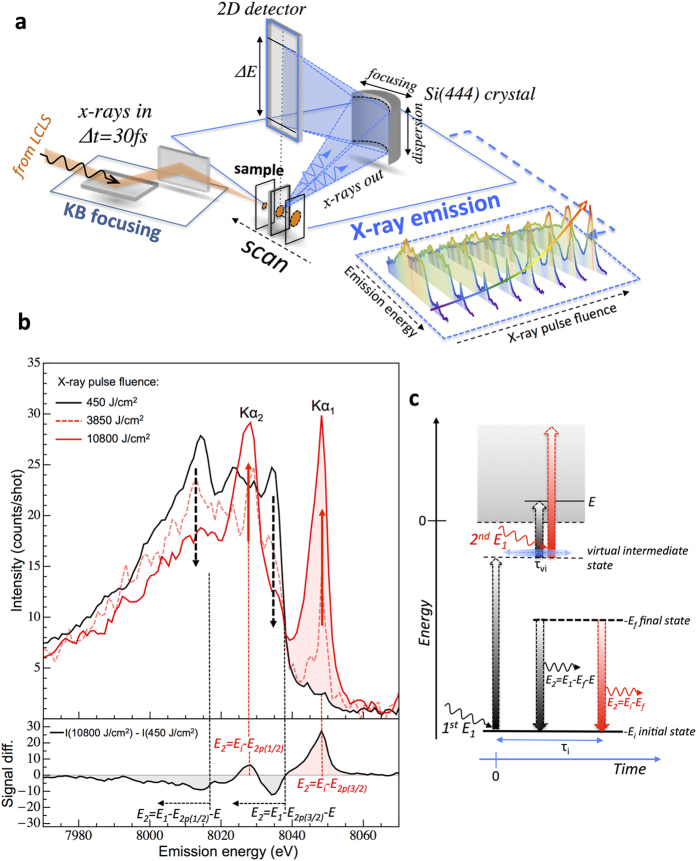
Experimental setup for nonlinear two-photon X-ray absorption spectroscopy. (**a**) schematics of the experimental setup showing the Kirkpatrick-Baez (KB) mirror focusing scheme and high energy resolution X-ray emission geometry. The X-ray emission data were recorded for different X-ray pulse fluence by moving the sample along the focus direction of the KB mirrors. (**b**) High energy resolution X-ray emission spectra recorded at an energy 12 eV below the K-shell ionization threshold of Cu for different incident X-ray fluences. The directions of spectral intensity changes with increasing X-ray pulse fluence are marked by black-dashed (HEROS) and red arrows (Kα emission), respectively. The spectrum difference between highest and lowest X-ray pulse fluence is plotted in bottom panel. (**c**) Schematic representation of radiative OPA (black arrows) and TPA (red arrows) processes in the off-resonant regime (*E*_*1*_* < E*_*i*_). While for the OPA process the emitted X-ray energy (*E*_*2*_) relates directly to the incoming X-ray energy (*E*_*1*_), the TPA mechanism leads to an ionization event and therefore the emitted X-ray energy is constant and equal to the energy difference between the initial (*E*_*i*_) and final (*E*_*f*_) electronic states. The *E*_*i*_ and *E*_*f*_ correspond to absolute values of the electron binding energies, and *E* is the energy of the photo-excited electron. The virtual intermediate state and the initial state are characterized by lifetimes marked by *τ*_*vi*_ and *τ*_*i*_, respectively.

In the present work, we used high energy resolution off-resonant X-ray emission spectroscopy^26^,^27^,^28^,^29^ (HEROS), which measures the X-ray emission from a sample while using an incidence X-ray energy set below a core level ionization threshold, to detect the ratio between OPA and TPA signals generated by ultra-short hard X-ray pulses in metallic copper as a function of X-ray fluence. The experiment was performed at the Coherent X-ray Imaging instrument^30^ at the Linac Coherent Light Source (Menlo Park, USA) XFEL (Fig. 1a, see Supplementary Information for details). In Fig. 1b we plot the X-ray emission spectra recorded for an incident X-ray photon energy tuned to 12 eV (E_1_ = 8967 eV) below the K-shell ionization threshold of Cu (E_i_ = 8979 eV), for different X-ray fluences. As can be noticed from Fig. 1b, as the X-ray fluence increases, large spectral variations are observed. The HEROS spectrum recorded at the lowest X-ray fluence of 450 J/cm^2^ (black curve) exhibits typical Raman-like features expected for an OPA mechanism. The high-energy cut-off around 8035 eV arises from energy conservation of the scattering process. This energy is defined as the difference between the energy of the incoming X-ray (E_1_) and energy of the final state (2p_3/2_, 933 eV)^31^. At lower emission energies, several features are detected that relate to the density of unoccupied electronic states of Cu, as discussed in detail in ref.^29^. For higher X-ray pulse fluences, two additional X-ray emission signals are detected at energies of 8048 eV and 8028 eV that correspond to the Kα_1_ and Kα_2_ X-ray emission lines, respectively. Due to the fact that the incoming beam energy was set below the K-ionization threshold of Cu, the detection of the Kα_1,2_ lines may be explained only by considering a two-photon electronic absorption process. In contrast to the OPA process in the off-resonant regime, for which the position of the HEROS spectrum is strictly defined by the incoming photon energy, the TPA mechanism leads to ionization and therefore the subsequent decay transition from final to initial core-states is independent of the incident X-ray photon energy. Thus, for an incoming beam energy set below the ionization threshold, the non-resonant Kα_1,2_ emission may be observed only as a result of a two photon X-ray absorption process (Fig. 1c). We should emphasize that the contribution from higher XFEL harmonics (e.g. 2^nd^ and 3^rd^) to the experimental data is negligible. Actually, at low X-ray fluences (e.g., 450 J/cm^2^) no contribution from the Kα_1,2_ emission could be observed when the XFEL beam was tuned below the K absorption edge. In addition the measured widths of the elastically scattered X-rays during the self-seeding operation were found to be about 0.75 eV (sigma) so that the incidence X-ray energy (8967 eV) of the self-seeded beam was 16 sigma below the 1s ionization threshold (see Supplementary Information). Actually, these elastically scattered signals were employed to sort the data shot-to-shot to ensure that the HEROS/TPA data used in the analysis corresponded to only well self-seeded pulses characterized by narrow spectral widths.

Moreover, we should note that any other photoionization paths of higher electronic levels (like L- or M-shells) of the atom would lead to a shift of the K-edge ionization energy to higher energies^32^, requiring therefore higher incidence X-ray energies in order to ionize the atom. The same statement holds for secondary ionization by photoelectrons and Auger electrons generated in the sample during the course of XFEL pulse. Assuming the most probable processes, such as L-shell ionization (with a cross-section of about 3.8 × 10^−5^ Å^2^/atom) and the subsequent Auger transitions, photoelectrons of about 8000 eV and Auger electrons in the 700–900 eV range are generated in the sample. The energies of these electrons are below the K-shell ionization threshold of the copper atom. Finally we note that the thermalization process of energetic electrons is expected to occur on time scales of a few tens to hundreds of fs^33^, which are longer than the 30 fs X-ray pulse duration employed in the present experiments.

Spectral analysis of the data presented in Fig. 1b reveals the decrease of HEROS signals at specific X-ray emission energies and the simultaneous growth of the non-resonant Kα_1,2_ signal intensity as the incident X-ray fluence increases. The observed spectral changes of the OPA and TPA signals can be explained by the assumption that the OPA excitation process at off-resonant conditions is achieved via the population of a virtual electronic state. Indeed, the off-resonant OPA process can be described as an electronic excitation with a virtual intermediate, mediated by the lifetime of the 1s core-hole state, with the intermediate state decaying by a radiative electronic transition (two-step process). This intermediate virtual state can also act as the first step in a TPA process. Therefore, at increasing X-ray fluence the OPA decay channel becomes depleted by the second photon-electron interaction. As a result, the OPA signal decreases, with a simultaneous increase of the TPA signal, suggesting a pure sequential two-photon absorption process.

**Figure 2:**
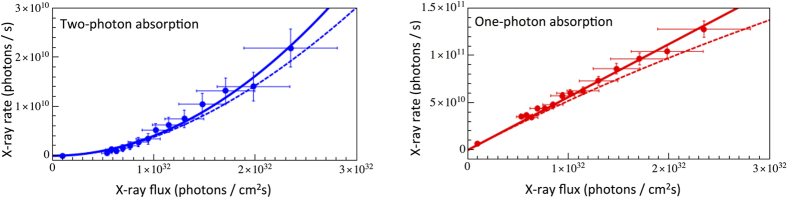
Determined X-ray transition rates at incidence X-ray energy of 8967 eV as a function of applied X-ray flux for TPA and OPA signals. Blue and red solid lines represent the quadratic/linear function fits to the experimental data. The dashed lines correspond to calculated rates using a model for a three-level system (for details see text).

A direct measure of the nonlinearity of a process is reflected in a square dependence of the measured X-ray signals as a function of the applied X-ray flux (*I*). In Fig. 2, we plot such dependence obtained from the experimental data and expressed in absolute X-ray transition rates per second for TPA (blue) and OPA (red) processes (see Supplementary Information for details). The observed square-dependence for TPA rates supports the observation drawn from Fig. 1b: the detection of non-resonant Kα_1,2_ emission demonstrates the presence of a nonlinear two photon X-ray absorption process. At the highest applied X-ray flux the TPA signal contribution is significant and amounts to about 15% of the total rate. The measured experimental data were fitted with quadratic and linear functions for TPA and OPA signals, respectively. Since the measured intensities are expressed as absolute X-ray transition rates, the fit is described by *R*_*TPA*_* = σ*_*TPA*_ * I^*2*^ for TPA^34^ and *R*_*OPA*_* = σ*_*OPA*_ * I for OPA, allowing thus for direct *σ*_*TPA*_ and *σ*_*OPA*_ cross-section determination. From the fits we obtain a TPA cross-section value of 4.1 (+/−1.1) × 10^−55 ^cm^4^s. For the OPA process a value of 5.6(+/−0.6) × 10^−22 ^cm^2^ is obtained, in agreement with other experimental data^35^.

As a direct consequence of the sequential TPA process, the sequential TPA yields will depend, not only on the incoming X-ray fluence, but also on the incoming X-ray energy. In the case of an OPA process under off-resonance conditions, which has to be regarded as the first step in a sequential TPA mechanism, the differential cross-sections may be described by a simplified formula resulting from the Kramers-Heisenberg relation^36^:


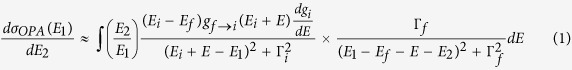


where *E*_*1*_ and *E*_*2*_ are the energies of the incoming and emitted X-rays and *E*_*i*_ and *E*_*f*_ are the energies of the initial and final electronic states. The factor *g*_*f*_ → _*i*_ accounts for decay rates from initial to final state, and *Γ*_*i*_, *Γ*_*f*_ are the inverse lifetime broadenings of the initial and final states respectively. *E* is the energy of the photo-excited electron and the *dg*_*i*_*/dE* function describes the density of unoccupied states, projected onto the initial state. We should note that the denominator in Eq. 1 sets the general trend of the OPA cross-section dependence. It may be assumed that the off-resonant scattering cross sections will vary as a function of the incident photon energy approximately as a Lorentz function with center at *E*_*i*_*−E*_*f*_ and width of *Γ*_*i*_. Thus, for decreasing values of the incident beam energy, the measured count rates in the HEROS spectra will be correspondingly lower.

**Figure 3:**
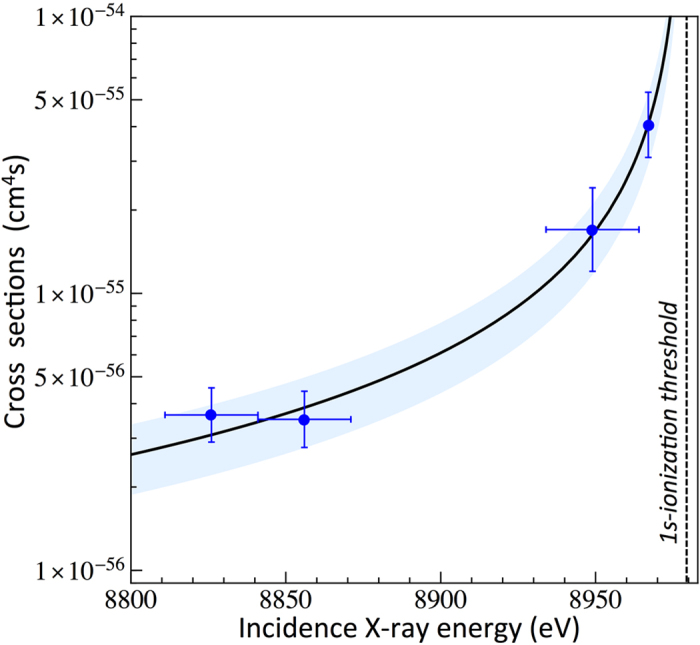
TPA cross-sections as a function of the incoming X-ray energy (blue circles). The line is a result of fitting the OPA cross-section dependence to experimental data points. The blue area represents the uncertainties related to the TPA cross section curve.

To probe the effect of the incident X-ray energy on the TPA yields, we recorded several X-ray emission spectra at different X-ray fluences and at different beam energies tuned down to 159 eV below the K-ionization threshold. At an incidence X-ray energy of 8967 eV (−12 eV detuning), we used the self-seeding mode of the LCLS machine, while at lower incidence X-ray energies SASE operation was employed in order to achieve higher X-ray peak powers. The SASE beam profiles were found to cover an energy range of +/−15 eV from the central X-ray energy so that the upper limit of the SASE beam energy profile was far below the 1s ionization threshold for each of the employed SASE beam energy (8949 eV, 8856 eV and 8826 eV). Moreover, as in the measurement using self-seeding operation, no contribution from the Kα_1,2_ emission was observed in the HEROS spectra collected in the SASE mode at low X-ray fluences (see Figure S2 of Supplementary Information). The detected Kα_1,2_ X-ray rates as a function of the X-ray flux were then used to determine the absolute cross sections for the TPA process and the obtained results are plotted in Fig. 3 (blue points) (see Supplementary Information for details). As shown, the TPA cross-sections exhibit a strong dependence on the incident X-ray energy, decreasing rapidly for larger detuning energies below the K-shell ionization threshold. The cross-sections on the level of 10^−56^–10^−55^ cm^4^s are obtained for incidence X-ray energies in the range of 8826–8970 eV (−159 to −12 eV detuning from the 1s ionization threshold). The TPA cross-section dependence on the incident X-ray energy was fitted with a theoretical curve describing the OPA cross-section (Eq. 1). Good agreement between the experimental data points and the theoretical curve is obtained, confirming our previous observations. The TPA yields strongly depend on the incident X-ray energy and follow the energy dependence relation for the OPA off-resonant process. In other words, in the off-resonant regime the OPA may be regarded as the first step in the sequential TPA mechanism, and as a consequence, the OPA cross-sections will be directly reflected in the measured TPA yields.

From the above fitting procedure the following relation between the OPA and TPA cross-section was obtained:


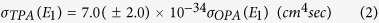


where *σ*_*OPA*_ is expressed in cm^2^ and *E*_*1*_ is the incident photon energy. The equation predicts a direct scaling between OPA and TPA processes. However, because of a lack of experimental data, it is difficult to judge to what extent the scaling factor of 7.0 × 10^−34^ (cm^2^s) may be applied as a general scaling rule between OPA and TPA mechanisms in the hard X-ray regime. However, we note that the obtained value is very close to the power-law value of 10^−33^ cm^n^s^n−1^ describing the ratio of multi-photon absorption cross sections at laser wavelengths^37^ (where *n* is the order of the multi-photon process). Based on Eq. 2 we also note, that the sequential TPA rates should exhibit saturation effects for condition 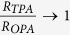 at an X-ray flux of 1.4 × 10^33^ photons/(cm^2^s) (i.e. the inverse of the scaling factor of 7.0 × 10^−34^ cm^2^sec). In order to compare the obtained results with other literature cross-sections for TPA processes (especially for low-Z elements), we first approximate *σ*_*OPA*_ using a *Z*-dependence based on an analytical solution of Eq. 1^38^. For hydrogen-like atoms, the energies and the atomic linewidth (*Γ*_*i*_) are proportional to *Z*^*2*^ while the oscillator strength *g*_*fi*_ scales^39^ as *Z*^*−1*^. The *dg*_*i*_*/dE* term is proportional to the 1s ionization cross-section, which may be expressed by^40^ *Z*^*4*^*/E*_*1*_^*3.5*^, giving a dependence of *Z*^*−3*^ for *E*_*1*_ when close to *E*_*i*_. Therefore, the general dependence for *σ*_*OPA*_ may be expressed as *Z*^*−4*^. Furthermore, based on our experimental results presented in Fig. 3, we introduce a term *E*_*i*_*/*(*E*_*i*_*−E*_*1*_), resulting from Kramers-Heisenberg theorem, that accounts for the incident energy dependence of *σ*_*OPA*_ under off-resonant conditions. Using the data presented in Fig. 3, we determined *Z* and *E*_*1*_ dependence of TPA cross-sections as:


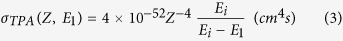


We should note that the above approximation does not account for resonance-effects in the TPA process and therefore may be used as estimate for TPA cross-sections at incidence energies, which are non-resonant with the initial and final electronic states. For helium, we obtain a *σ*_*TPA*_ of 1.4 × 10^−52^ cm^4^s, which compares with the experimental result^41^ of 2.5 × 10^−52^ cm^4^s at an incidence energy of 20.19 eV. For higher *Z* elements, the reported XFEL Ne^8+^ experimental TPA cross-sections^11^ of 7 × 10^−54^ cm^4^s were examined with time-dependent calculations^42^ that yield *σ*_*TPA*_ of 1.6 × 10^−55^ cm^4^s. For *E*_*i*_ = 1196 eV and *E*_*1*_ = 11_*1*_0 eV, Eq. 3 gives an approximate value for *σ*_*TPA*_ of 5.6 × 10^−55^ cm^4^s, which is in good agreement with the theoretical results and is about 10 times lower than the reported experimental value. Finally, Tamasaku *et al*.^10^ recently reported a TPA cross-section of 6.4 × 10^−60^ cm^4^s for Ge at very large detuning energies from the ionization threshold (*E*_*1*_ = 5600 eV, *E*_*i*_ = 11103 eV). Equation 3 predicts a value for *σ*_*TPA*_ of 7.7 × 10^−58^ cm^4^s. At present, it is difficult to judge the reason for some of the discrepancies, which may originate from either approximations made within Eq. 3 (i.e. neglecting relativistic effects) or from experimental uncertainties in the XFEL TPA cross-section measurements, such as the pulse duration or the time-dependent pulse structure^42^,^43^. At present, because of a lack of experimental and theoretical data, it is hard to determine the level of correction for the relativistic effects on the TPA cross sections. In case of above edge single photoionization, the quantum mechanics calculations for hydrogenic and non-hydrogenic states show that multi-electron correlation effects have to be considered for precise cross-sections determination^44^. Since the relativistic effects are expected to become non-negligible for mid-Z and high-Z elements, the generalized cross-section presented in Eq. 3 should account for these effects, which vary as a function of the atomic number Z of the material. Nonetheless, using Eq. 3 may be regarded as a rough order-of-magnitude estimate of the TPA cross-sections.

The present experimental approach allows the simultaneous probing of the intermediate and final state populations during the course of the TPA process. The TPA cross-section may be described as a product of the cross-sections of each absorption event with the lifetime of the virtual intermediate state^45^: *σ*_*TPA*_* = σ*^(*1*)^*τ*_*vi*_*σ*^(*2*)^. Since *σ*^(*1*)^ corresponds to the OPA cross-section for off-resonant excitation and *σ*^(*2*)^ to the cross-section for the second X-ray absorption step, both values may be quantitatively compared based on our experimental data. Indeed, we should note that the experimentally established scaling factor in Eq. 2 of 7.0 × 10^−34^ (cm^2^sec) corresponds to *τ*_*vi*_*σ*^(*2*)^. At off-resonant conditions, the effective scattering time depends on the detuning energy^46^ i.e.: 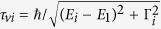, where *Γ*_*i*_ is the width of the initial state. Using a width for the Cu K-shell of 1.49 eV^32^, we obtain 5.4 × 10^−17^s for *τ*_*vi*_ at *E*_1_ of 8967 eV. The calculated cross-section for the second X-ray absorption step *σ*^(*2*)^ is thus found to be 1.3 × 10^−17^ cm^2^. It is interesting to note that this value is five orders of magnitude higher than the OPA off-resonant cross-sections *σ*^(*1*)^ of 5.6 × 10^−22^ cm^2^. This result supports our previous observations: the yields for sequential TPA processes are dominated by the cross-section for the first absorption step.

The measured OPA and TPA X-ray rates were tested with a rate equation model assuming a three-level electronic system (see Supplementary Information for derivation). For the calculations we used the experimentally-determined cross-sections for the first *σ*^(*1*)^ and second *σ*^(*2*)^ absorption steps. The computed rates are plotted in Fig. 2 as blue for TPA and as red for OPA dashed lines, respectively. As shown, the calculated X-ray rates follow well the general dependence for both OPA and TPA processes. We would like to stress that using a simplified rate equation model allows the X-ray rate dependences to be estimated with good accuracy, confirming that the higher order effects, such as direct L-shell ionization and electron rearrangements, distort the measured signals in a negligible way. Indeed, the observed 10% discrepancy between the measured data and calculated curves, though still within the measurement errors, may indicate that for higher X-ray fluxes the three-level rate equation model would have to be extended to account for other X-ray interaction processes and subsequent electronic transitions.

## Summary

Based on these results, we will now discuss several important aspects of the TPA mechanism and its application to nonlinear X-ray spectroscopy. First, we note the relatively large contribution of the TPA signal at energies close to the ionization threshold. In this experiment we employed a fluence of a few thousands of J/cm^2^, which corresponds to an X-ray flux of about 10^11^–10^12^ X-rays in a 1–4 μm spot, a value easily achievable during XFEL experiments. As a consequence, in such an X-ray regime the sample may undergo strong nonlinear processes. Therefore, the X-ray fluence threshold for TPA is of primary importance for many XFEL experiments in which nonlinear X-ray processes should be avoided for accurate data interpretation. The transition regime between the OPA and TPA mechanisms is also of importance for “probe before destroy” experiments at XFEL. Therefore, further studies of X-ray fluence thresholds versus incoming X-ray energy are of importance in order to recognize the regimes at which the TPA mechanism becomes important.

Finally, in analogy with TPA mechanisms at optical laser wavelengths, hard X-ray TPA spectroscopy may be foreseen to be a novel spectroscopy tool, thanks to the different selection rules when compared to the OPA process. For X-ray Absorption Near Edge Spectroscopy (XANES) on 3d elements, the TPA-XANES experiments would allow to enhance the weak pre-edge features observed in typical synchrotron experiments. The transitions corresponding to these pre-edge structures are of importance because they provide information on the symmetry and electronic environment of the scattering atom, and have been used for many scientific applications. The TPA process on pre-edge transitions would allow further testing of theoretical approaches for XANES measurements, providing more details on the hybridization of the final state (e.g. 3d-2p in metal oxides) and of the dipole and quadrupole contributions to these states. For example one would expect an enhancement in the transition probability for a quadrupole-allowed peak. In the case of Extended X-ray Fine Structure Absorption Spectroscopy (EXAFS) we do not expect any significant changes as compared to OPA EXAFS. The photoelectron ejected from the atom by the TPA process will undergo single-scattering paths on the surrounding atoms in the same way as in case of the OPA mechanism. However, the real application of TPA is challenging and needs to be further explored. For example, in order to access the *1s* → *3d, 4d* transitions, which are found in the pre-edge regions of the K-edge X-ray absorption spectra of 3d and 4d metals, the incident X-ray energy has to be detuned to an energy half that of the *1s* ionization threshold. Because of the strong incident energy dependence of the TPA cross-sections, at such large detuning energies from the 1s ionization threshold, the TPA yields become very small. However, based on our results, we foresee an alternative application of hard X-ray TPA spectroscopy in X-ray-optical wave mixing experiments. Recently, the mixing of X-ray and laser pulses was measured in a diffraction experiment, demonstrating the ability of performing X-ray/optical sum-frequency generation^15^. This general approach of mixing optical and X-ray pulses in a sample can also be applied to TPA spectroscopic experiments. In these experiments, instead of tuning the incoming X-ray photon energy to an energy half that of the excitation energy, the X-ray energy may be set close to the ionization threshold, where sequential TPA cross-sections become relatively large. Then by mixing the optical laser pulses with the X-rays exciting the sample, the missing energy may be added to the intermediate virtual state allowing quadrupole- or forbidden-excitations to be directly probed.

## Additional Information

**How to cite this article**: Szlachetko, J. *et al*. Establishing nonlinearity thresholds with ultraintense X-ray pulses. *Sci. Rep.* **6**, 33292; doi: 10.1038/srep33292 (2016).

## Supplementary Material

Supplementary Information
