# Supplementary material for: Establishing nonlinearity thresholds with ultraintense X-ray pulses
Source: Sci Rep. 2016 Sep 13;6:33292. doi: 10.1038/srep33292 (PMC5020491; doi:10.1038/srep33292)
Supplement: Supplementary Information [file srep33292-s1.pdf]

Supplementary Information: Establishing nonlinearity thresholds with ultraintense X-ray pulses

Jakub Szlachetko[1,2,*], Joanna Hoszowska[3], Jean-Claude Dousse[3], Maarten Nachtegaal[1], Wojciech Błachucki[3], Yves Kayser[1], Jacinto Sà[4,5], Marc Messerschmidt[6,7], Sebastien Boutet[6], Garth J. Williams[6,8], Christian David[1], Grigory Smolentsev[1], Jeroen A. van Bokhoven[1,9], Bruce D. Patterson[1], Thomas J. Penfold[1], Gregor Knopp[1], Marek Pajek[2], Rafael Abela[1], Christopher J. Milne[1,*]

[1]Paul Scherrer Institut, Villigen, Switzerland.
[2]Institute of Physics, Jan Kochanowski University, Kielce, Poland.
[3]Department of Physics, University of Fribourg, Fribourg, Switzerland.
[4]Department of Chemistry, Uppsala University, Uppsala, Sweden.
[5]Institute of Physical Chemistry, Polish Academy of Sciences, Warsaw, Poland.
[6]Linac Coherent Light Source, SLAC National Accelerator Laboratory, Menlo Park, USA.
[7]NSF BioXFEL STC, 700 Ellicott Street, 14203 Buffalo, USA.
[8]Brookhaven National Laboratory, Upton NY 11973 USA.
[9]Institute for Chemical and Bioengineering, ETH Zürich, Zürich, Switzerland.
* jakub.szlachetko@psi.ch, jszlachetko@ujk.edu.pl, chris.milne@psi.ch.

S1. Experiment.

In the present work, we used high energy resolution off-resonant X-ray emission spectroscopy[1-4] (HEROS), which measures the X-ray emission from a sample while using an incidence X-ray energy set below a core level ionization threshold, to detect the ratio between OPA and TPA signals generated by ultra-short hard X-ray pulses in metallic copper as a function of X-ray fluence. The experiment was performed at the Coherent X-ray Imaging instrument[5] at the Linac Coherent Light Source (Menlo Park, USA) XFEL. During the experiment we employed both self-seeded[6] and self-amplified spontaneous emission (SASE) operation modes of the LCLS XFEL, allowing to some extent, to have control over the X-ray beam monochromaticity and X-ray peak power. In order to achieve high X-ray fluence, the incoming X-ray beam was focused by a set of Kirkpatrick-Baez mirrors to a spot size below 0.5 μm x 0.5 μm (FWHM). The incident X-ray photon energy was tuned around the Cu K-absorption edge (E_i=8979 eV) and the LCLS source was operated at a repetition rate of 120 Hz. The machine delivered 30 fs duration X-ray pulses in the high charge mode (250 pC) with a typical X-ray flux of ~10^{11} X-rays/pulse in self-seeded mode and ~10^{12} X-rays/pulse during SASE operation. The sample was continuously moved during measurement in order to avoid multiple X-ray pulse hits on the same spot. The X-ray fluence was adjusted by placing the sample at different positions along the beam propagation direction. In this way, the effective spot size was varied up to 10 μm x 10 μm, which in combination with SASE/self-seeding operation allowed the X-ray fluence to be varied by over two orders of magnitude. The X-ray spot size was measured off-line using beam imprints[7] on a gold-coated glass substrate. The X-ray fluence used in the experiment was in the range of 10^2 J/cm^2 to 10^3 J/cm^2 for seeded operation and up to 10^5 J/cm^2 for SASE operation. For X-ray emission detection at around 8040eV ($K\alpha_{1,2}$ X-ray lines, $2p_{3/2}\rightarrow 1s$ and $2p_{1/2}\rightarrow 1s$ transitions) we employed a spectrometer

operated in a dispersive von Hamos geometry using a Si(444) crystal at a radius of 25 cm and a 140K CSPAD detector[8]. The spectrometer was arranged in a vertical scattering geometry and at close to 90-degree scattering angle with respect to the incoming beam. The chosen spectrometer geometry allowed us to record X-ray emission spectra on a shot-to-shot basis, covering about 100 eV emission energy range for every X-ray pulse. Because of the relatively large Bragg angle for Si(444) diffraction (around 80°) and the use of a segmented-type crystal[9] the spectrometer resolution was about 0.5 eV. This resolution is 1-3 times better than the natural lifetime broadening of the Cu 1s, $2p_{3/2}$ and $2p_{1/2}$ electronic levels (1.49eV, 0.61eV and 1.04eV[10], respectively).

S2. Determination of absolute X-ray rates

The non-resonant $K\alpha_{1,2}$ X-ray emission spectrum, plotted in Figure S1, was used to determine the experimental efficiency. At low X-ray fluences (<400J/cm^2) and incidence X-ray energies above the ionization threshold (9077eV) the measured integrated $K\alpha_{1,2}$ intensity per incident X-ray pulse is expressed as follows:

$$N_{K\alpha(OPA)} = \Omega \cdot \sigma_K \cdot n \cdot x \cdot c_{abs} \cdot \omega_{K\alpha} \cdot N_0 \qquad (1)$$

where Ω is the factor accounting for experimental efficiency, σ_K is the K-shell ionization cross-section, n is the number of atoms per unit volume, c_{abs} is the correction for self-absorption, $\omega_{K\alpha}$ is the fluorescence yield and N_0 is the number of X-rays on the sample per pulse. C_{abs} was calculated with a well known formalism[11] accounting for the incidence and exit angles on the sample which were both 45°in our experiment. Based on Eq.S1 and using tabulated values[12,13] of σ_K and $\omega_{K\alpha}$ an experimental efficiency Ω of 1.52x10^{-7} was obtained.

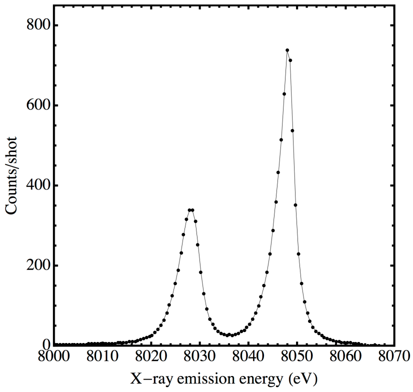

Figure S1: The non-resonant $K\alpha_{1,2}$ X-ray emission measured at an incident beam energy of 9077eV and incoming X-ray intensity of 3.35x10^{11} x-rays/pulse. The spectrum represents the average of 2600 shots.

X-ray rates for one-photon absorption are defined by $R_{OPA}[s^{-1}]=\sigma_{OPA}I$ and the rates for two-photon absorption by $R_{TPA}[s^{-1}]=\sigma_{TPA}I^2$, where I is the x-ray flux (photons/(cm^{2}s)). Therefore, using Eq.1 the absolute rates for OPA/TPA signals at an incidence energy tuned below the ionization threshold may be calculated as follows:

$$R_{OPA}(s^{-1}) = \frac{N_{OPA}}{\Omega \cdot n \cdot x \cdot \omega_{K\alpha} \cdot c_{abs} \cdot 2\pi \cdot \sigma_{x,y} \cdot \sigma_t} \quad (2a)$$

$$R_{TPA}(s^{-1}) = \frac{N_{TPA}}{\Omega \cdot n \cdot x \cdot \omega_{K\alpha} \cdot c_{abs} \cdot 2\pi \cdot \sigma_{x,y} \cdot \sigma_t} \quad (2b)$$

Since all the measured signals involve the 2p→1s decay we can thus assume that the fluorescence yield is the same for non-resonant emission, off-resonant OPA and TPA processes. The $\sigma_{x,y}$ and σ_t accounts for the Gauss-like distributions of the spatial beam profile on the sample and time profile of the incident X-ray pulses.

S3. X-ray emission spectra at different detuning energies

In figure S2 we plot X-ray emission spectra recorded for different incoming X-ray energies tuned below the K-ionization threshold at low (black) and high (blue) X-ray fluences. The incident X-ray energies employed in the experiment were chosen to avoid any possible internal 1s to 3p resonance transitions at 8905eV that could interfere with the measured TPA yields. The spectra reveal the presence of the $K\alpha_{1,2}$ X-ray emission only when a high enough X-ray fluence is applied. Thus, the contribution from higher XFEL harmonics is assumed to be negligible. In order to check the spectral profile of the incident X-rays during the self-seeded operation of LCLS, the elastically scattered X-rays from the sample were measured with the von Hamos spectrometer at the same time as the HEROS/TPA spectra. The elastic scattering was measured using a Ge(880) crystal covering the required energy range of the incoming beam while the HEROS/TPA X-rays were diffracted by Si(444) crystal. The elastic scattering data presented in Figure S3 were fitted with a Gaussian profile to determine the width of the incoming beam. A sigma value of 0.75eV was obtained.

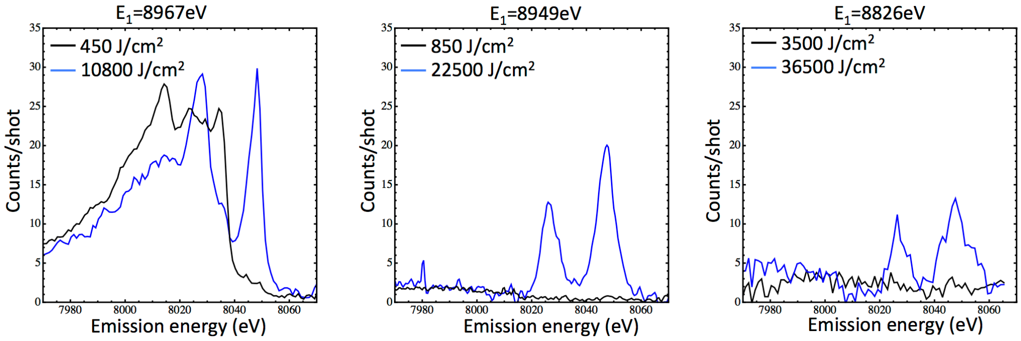

Figure S2: X-ray emission spectra recorded at different incoming X-ray energies and X-ray fluences.

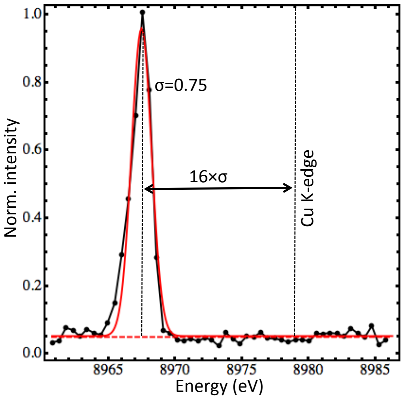

Figure S3: Measured spectrum corresponding to the elastic scattering of the self-seeded LCLS pulses (black line). The spectrum represents the sum of all shots used in the analysis of the HEROS/TPA data measured at 8967 eV (see Figure 1b). For the determination of the beam profile width, the elastic scattering spectrum was fitted with a Gaussian profile (red line). From the fit, a standard deviation σ = 0.75eV was found.

The experimental N_{TPA} intensities were obtained from the integration of the experimental spectra over the $K\alpha_{1,2}$ range (8020eV-8060eV) and by subtracting the background which was determined by a linear fit of the counts under the $K\alpha_{1,2}$ signal. For the incidence energy of 8967eV which is close to the ionization threshold so that the TPA signal is partially overlapping with the OPA signal, we determined first the $K\alpha_1$ counts by integrating the spectrum over the energy range 8040eV-8060eV and assuming for the background intensity the value determined from the X-ray emission spectrum measured at the lowest fluence, i.e., without contribution of TPA. The OPA intensity was then obtained by subtracting the TPA contribution from the total integrated spectral intensity. A $K\alpha_2$-to-$K\alpha_1$ intensity ratio of 51% was assumed. Finally, the rates for the OPA process presented in Figure 2 of the manuscript were further corrected to account for the intensity of the x-rays emitted below 7970eV, i.e., the low energy limit of our experimental setup. Since the energy range of the HEROS x-rays extends down to 0eV, the Eq.1 of the manuscript was employed to determine the missing HEROS yields. A value of 35% was found. The so obtained N_{OPA} and N_{TPA} intensities were then used to calculate absolute rates using Eq.2a and Eq.2b. The corresponding results are plotted for three different incidence X-ray energies in Figure S4.

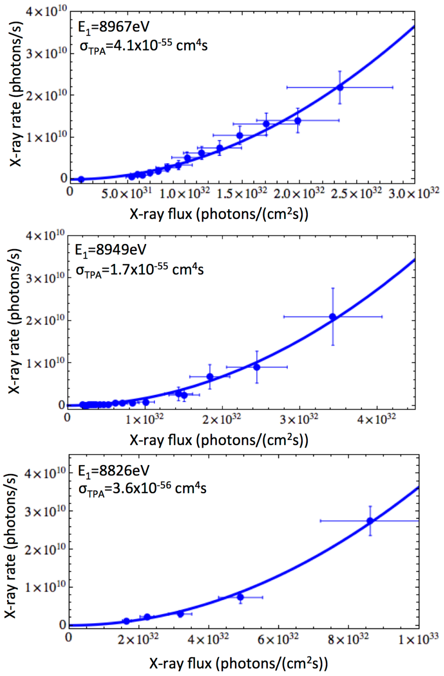

Figure S4: Determined TPA rates at different incidence energies below the K-ionization threshold. The TPA cross sections were obtained from a square function fit to the experimental data.

S4. Rate equations

A simplified three levels system was assumed for the rate equations modeling of the sequential two-photon absorption. A schematic drawing showing the electronic transitions is presented in Figure S5.

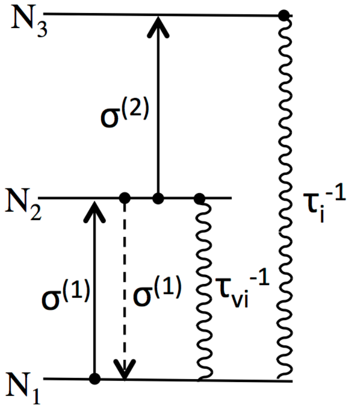

Figure S5: Schematic representation of the three levels system used for the rate equations model.

The following rate equations were considered:

$$\frac{dN_1}{dt} = -\sigma^{(1)} \cdot I \cdot (N_1 - N_2) + \frac{N_2}{\tau_{vi}} + \frac{N_3}{\tau_i} \qquad (3)$$

$$\frac{dN_2}{dt} = \sigma^{(1)} \cdot I \cdot (N_1 - N_2) - \sigma^{(2)} \cdot I \cdot N_2 - \frac{N_2}{\tau_{vi}} \qquad (4)$$

$$\frac{dN_3}{dt} = \sigma^{(2)} \cdot I \cdot N_2 - \frac{N_3}{\tau_i} \quad (5)$$

$$N_1 + N_2 + N_3 = N = 1. \quad (6)$$

where N_1 is the population of the ground state, N_2 the population of the virtual intermediate state and N_3 the population of continuum states of atoms (ionized states) through the second X-ray absorption. In above equations we neglect the direct TPA absorption (i.e. simultaneous two X-ray absorption) as this process is expected to be three orders of magnitude lower than sequential TPA[14]. The incident X-ray flux is represented by I in photons/(cm^{2}s) and $\sigma^{(1)}$, $\sigma^{(2)}$ are the cross sections in cm^2 for the first and second absorption step, respectively. Since the experiment was performed at conditions where the pulse length Δt was much longer then the lifetimes τ_{vi} and τ_i, we assumed a stationary case with $\dot{N}_1 = \dot{N}_2 = \dot{N}_3 = 0$, with boundary condition $N_1(t = 0) = N$ .The equations (3-6) can be solved for N_2 and N_3, and the rates for OPA and TPA may then be calculated with the following formulas:

$$R_{OPA}(s^{-1}) = \frac{N_2}{\tau_{vi}} = \frac{I\sigma^{(1)}N}{1+2I\sigma^{(1)}\tau_{vi}+I\sigma^{(2)}\tau_i+I^2\sigma^{(1)}\sigma^{(2)}\tau_{vi}\tau_i} \quad (7)$$

$$R_{TPA}(s^{-1}) = \frac{N_3}{\tau_i} = \frac{I^2\sigma^{(1)}\sigma^{(2)}\tau_{vi}N}{1+2I\sigma^{(1)}\tau_{vi}+I\sigma^{(2)}\tau_i+I^2\sigma^{(1)}\sigma^{(2)}\tau_{vi}\tau_i} \quad (8)$$

References:

1. Szlachetko, J. *et al.* High energy resolution off-resonant spectroscopy at sub-second time resolution: (Pt(acac) 2) decomposition. *Chem. Commun.* **48,** 10898–10900 (2012).
2. Kavčič, M. *et al.* Hard x-ray absorption spectroscopy for pulsed sources. *Phys. Rev. B* **87,** 075106 (2013).
3. Milne, C. J., Penfold, T. J. & Chergui, M. Recent experimental and theoretical developments in time-resolved X-ray spectroscopies. *Coord. Chem. Rev.* **277,** 44–68 (2014).
4. Szlachetko, J. *et al.* The electronic structure of matter probed with a single femtosecond hard x-ray pulse. *Struct. Dyn.* **1,** 021101 (2014).
5. Liang, M. *et al.* The Coherent X-ray Imaging instrument at the Linac Coherent Light Source. *J. Synchr. Rad.* **22,** 514-519 (2015).
6. Amann, J. *et al.* Demonstration of self-seeding in a hard-X-ray free-electron laser. *Nature Photon* **6,** 693–698 (2012).
7. David, C. *et al.* Nanofocusing of hard X-ray free electron laser pulses using diamond based Fresnel zone plates. *Sci. Rep.* **1,** 1–5 (2011).
8. Herrmann, S. *et al.* CSPAD-140k: A versatile detector for LCLS experiments. *Nucl. Instr. Meth. Phys. Res. A* **718,** 550–553 (2013).
9. Szlachetko, J. *et al.* A von Hamos x-ray spectrometer based on a segmented-type diffraction crystal for single-shot x-ray emission spectroscopy and time-resolved resonant inelastic x-ray scattering studies. *Rev. Sci. Instrum.* **83,** 103105–103105–7 (2012).
10. Campbell, J. L. & Papp, T. Widths of the atomic K-N7 levels. *Atomic Data and Nuclear Data Tables* **77,** 1–56 (2001).
11. Sharma. *Atomic And Nuclear Physics*. (Pearson Education India, 2008).
12. Berger *et al.* XCOM: Photon Cross Section Database (version 1.5). at <http://physics.nist.gov/xcom >
13. Krause, M. O. Atomic radiative and radiationless yields for K and L shells. *J Phys Chem Ref Data* **8,** 307–327 (1979).
14. Tamasaku, K. *et al.* X-ray two-photon absorption competing against single and sequential multiphoton processes. *Nature Photon* **8,** 313–316 (2014).
